# How to Care for the Insane

**Published:** 1886-06

**Authors:** 


**How to Care for the Insane.** A Manual for Attendants in Insane Asylums. By WILLIAM D. GRANGER, M. D., First Assistant Physician Buffalo State Asylum for the Insane, Buffalo, N. Y. New York and London: G. P. Putnam's Sons. 1886.

In 1883, there was established at the Buffalo State Asylum one of the first training schools for attendants, and last month we were pleased to present to our readers a report of the first commencement exercises. It is fitting that the one who has been the prime mover in this matter should, at this time, present to the public a manual on this subject. "The writer believes that all attendants should be regularly instructed in their duties, and the highest standard of care can be reached only when this is done." This manual aims to impart this instruction, and, if studied, it certainly will be a useful and necessary book for every asylum employee. It is well written, being plain and comprehensive. The subject seems to be well covered. The topographical work is in keeping with the general style of the book. It is certainly a credit to the Doctor and the publishers, and the work should have a large sale, thereby being a great aid to every one interested in the care of these most unfortunate persons.

